# Increased Responsibility and Transparency in an Era of Increased Visibility

**DOI:** 10.1371/journal.pmed.1000364

**Published:** 2010-10-26

**Authors:** The PLoS Medicine Editors

## Abstract

The PLoS Medicine editors discuss further the paper by Peter Gøtzsche and colleagues on journals' competing interests. The editorial reinforces the call by Harvey Marcovitch for journals to be transparent and thus discloses PLoS Medicine's sources of income for 2009.

In the global information age, with more and more tools available to disseminate health information, medical journals still occupy a unique position of influence as trusted sources of information—clearly different from the vast majority of medical information produced and disseminated daily.

But along with this influence comes a unique set of responsibilities. Medical journals are expected to be impartial arbiters of the research submitted to them. However, the pressures on journals to publish papers have increased year after year as the rewards to authors, their institutions, and the funders of the work for publication increase. Aware of such external pressures, journals now rightly expect authors to consider and declare all potential sources of conflict [1]. Perhaps less well understood, or at least discussed, is that journals themselves also experience pressure—to be profitable, or at least self-sustaining in order to survive; and to maintain and even enhance the journal's reputation within communities of authors, readers, and other journals.

In a paper published this week in *PLoS Medicine* the potential influence of some of these pressures on journals are examined. Andreas Lundh and colleagues [2] examined clinical trial reports in six general medical journals over two time periods. (Note: this analysis does not include *PLoS Medicine* as it did not launch until after the first time period.) The authors asked whether there could be any association of the publication of these papers with the journals' finances or their calculated impact factor (not exactly the same as the impact factor released by Thomson ISI) and whether that association changed over time. The conclusion that publication of industry-supported trials is associated with a disproportionate increase in citations, and that sales of reprints from these papers are a substantial source of income for journals, is perhaps not surprising, but it is alarming.

There are, of course, caveats in this analysis—for example, the increased number of citations for industry-funded clinical trials may indicate that these papers were especially important trials. Nonetheless, the paper will not be comfortable reading for any editor of a biomedical journal. Even though there is no suggestion in this paper that any editorial decisions were made with an eye on reprint sales or citations, the analysis does show that the potential income, and the disproportionate number of citations from papers with a clear commercial interest, present a potential source of conflict for journals aside from any conflicts for authors or funders of these studies.

Much has been written about problems with reprint sales and impact factors [3]–[6]. This paper, by showing an association of industry-funded trials with citations and reprint sales, provides further evidence that these two pressures—unless handled carefully and transparently by journals—may lead to questions about whether such potential competing interests are acceptable. It should thus serve as a wake-up call for every journal that makes a substantial living from reprints to examine how they manage such potentials for conflict.

What are the defenses journals can use against these competing interests? The first is to promote a wide awareness of all the potential competing interests that can arise during publication, not just those of authors. Journals have been keen to publish on the potential misdemeanors of authors, but have been less interested in those of journals and publishers. Papers such as Lundh and colleagues' thus serve an important function in bringing such topics to debate. The second defense is for journals themselves to be transparent in their handling of their own potential financial and nonfinancial biases, which could stem from ownership relationships, publication models, and relationships with partner organizations, among others. There are already a number of editorial organizations that promote best practices in scholarly publishing [1],[7],[8]. Though mostly they deal with concerns about authors and reviewers, there is some guidance for editors on their own conflicts [9], though rarely about the journals' conflicts.

In the summary of their paper, Lundh and colleagues make a further, more radical suggestion—that medical journals publish their annual returns. We agree that this is appropriate. PLoS discloses its finances in a number of ways: In the 990 report we are required to publish as a nonprofit organization registered in the US and in our progress report [10]. In the interest of further transparency *PLoS Medicine*'s sources of income for 2009 are listed in the footnote to this article and we intend to place them each year on the page that currently lists the editors' competing interests [11]. Similarly, we'd argue that journals should consider whether the chasing of impact factors may also be a source of bias that needs to be recognized and declared. For us, we wish to make our relationship with the impact factor clear: Although the PLoS journals are all indexed by ISI and hence an impact factor is calculated for each, PLoS does not promote impact factors anywhere on its Web sites, and instead we urge authors and readers to assess the impact of individual articles via the article-level metrics we provide with each article [12].

The internet has spurred an intellectual revolution in the dissemination of medical information. Journals have thus far been accepted as one of the most trusted sources of information. It's clear, however, that in order to maintain that trust, journals and editors need to continue to consider all the pressures that can arise in publishing and put in place robust, transparent procedures for handling all the potential conflicts that can arise, whether they are those of authors, editors, or the journals themselves.
